# Neurophysiological Mechanisms of Cow–Calf Bonding in Buffalo and Other Farm Animals

**DOI:** 10.3390/ani11071968

**Published:** 2021-06-30

**Authors:** Agustín Orihuela, Daniel Mota-Rojas, Ana Strappini, Francesco Serrapica, Ada Braghieri, Patricia Mora-Medina, Fabio Napolitano

**Affiliations:** 1 Facultad de Ciencias Agropecuarias, Universidad Autónoma del Estado de Morelos, Cuernavaca 62209, Morelos, Mexico; 2 Neurophysiology, Behavior and Animal Welfare Assessment, DPAA, Universidad Autónoma Metropolitana, (UAM), Mexico City 04960, Mexico; dmota100@yahoo.com.mx; 3 Faculty of Veterinary Sciences, Animal Science Institute, Universidad Austral de Chile, Valdivia 5090000, Chile; anastrappini@uach.cl; 4 Dipartimento di Agraria, Universitàdi Napoli Federico II, Via Università100, 80055 Portici, Italy; francesco.serrapica@unina.it; 5 Scuola di Scienze Agrarie, Forestali, Alimentari ed Ambientali, Università degli Studi della Basilicata, 85100 Potenza, Italy; ada.braghieri@unibas.it (A.B.); fabio.napolitano@unibas.it (F.N.); 6 Livestock Science Department, Facultad de Estudios Superiores Cuautitlán, Universidad Nacional Autónoma de México (UNAM), Mexico City 54714, Mexico; mormed2001@yahoo.com.mx

**Keywords:** imprinting, bonding, animal welfare, mother–young relationship, buffaloes

## Abstract

**Simple Summary:**

The present paper reviews the importance of bonding for the survival and well-being in the cow–calf relationship. The review focuses on buffaloes and information from other species is used for comparison or to find more general patterns in the absence of specific sources. Differences between several farm species are also described, focusing on the role played by the sensory stimuli during the sensitive period after birth. How bonding can be classified according to the predominant senses used by different species, the importance of learning (i.e., imprinting) in the development of mother–young relationship, and the neurobiological mechanisms involved are also delineated. Finally, some examples of the main factors that can affect the mother–young relationship in the field are given. By understanding the imprinting at brain level, as well as the relationship with behavior, we gain a deeper insight into the critical role that experience, and environmental factors play in shaping the development of the mother–offspring bond.

**Abstract:**

In buffaloes and other mammalian farm species, the mother provides food and protection to the young, but she is also the main source of behavioral and social learning for the offspring. It is important that mother and young establish a bond based on a learning mechanism defined as “imprinting” early after parturition during the sensitive period, on which the welfare and survival of the offspring will depend. This review aims to summarize and discuss current knowledge regarding the imprinting process, the neurobiological pathways that are triggered during this sensitive period, and the development of the cow–calf bond. Touch, hearing, vision, and smell seem to be the predominant senses involved during imprinting in buffaloes and other mammalian farm species. In buffalo, bonding is very particular due to the expression of specific behaviors, such as allo-suckling and communal rearing. In general, imprinting and the subsequent bond may be affected by the lack of experience of the mothers or dystocic parturitions, which occur most frequently with male calves and in primiparous dams. The main problems in the development of this process include lack of seeking a protected and isolated place to give birth; moving from the birth-site after parturition; insufficient postpartum care; aversion or aggressiveness towards the newborn, or abandonment of the newborn. The process can develop differently according to the species. However, the correct development of the cow–calf relationship represents, regardless of the species, a key factor for their fitness.

## 1. Introduction

In most mammals, care for the young depends primarily on the mother [1]. She is the most important social contact for the newborn during the first months of life because, in addition to feeding and care, she provides the offspring with the acquisition of important information in relation to the physical and social environment [2,3]. Therefore, the bond that is generated at birth, which is dependent on a learning process known as “imprinting”, which allows the identification of the mother [4], is fundamental for the survival of the young. Imprinting is a learning mechanism which allows the rapid acquisition of a clear and stable preference for a particular type of stimulus to which the animal is exposed during a sensitive period [5,6].

A bonded mother overtly expresses maternal behavior, including suckling, and shows negative behavioral responses, such as increased vocalizations and locomotor activity, in response to separation from her young; responses that decrease after reunion [7,8]. Similarly, the bonded offspring express affiliative behavior toward the mother, and show marked distress when separated from her [8].

The period during which the bond is stablished is known as the “sensitive period”, since it is a limited stage dependent on early learning of sensory stimuli (sight, touch, smell, and hearing) exchanged naturally with the mother or the environment during the neonatal phase [2,3,4]. Any interference in this sensitive period, such as human intervention or some other external stimuli, could lead to the development of an inaccurate imprint and alterations of the bond. This can generate reproductive, social, or behavioral disturbances in the newborn, or the mother’s rejection of her offspring [9]. In fact, the imprinted bond with the mother can affect the sensory preferences of animals once they reach adulthood, including feeding, maternal and sexual behaviors [10,11]. An extreme example has been reported in small ruminants by Kendrick et al. [12], who showed that rams that were raised by goats, or goats that were raised by sheep, preferred females of the species that raised them, rather than females of their own species as a sexual partner when adults.

Altered imprinted bonds reduce the newborn’s probability of survival and impair their behavioral development to adulthood [13]. For example, aggressiveness can increase in animals reared in isolation from social groups, which is attributed to the fact that during early stages they did not receive the necessary information or stimulation from the mother and, consequently, did not learn how to react to or interact with conspecifics in situations needing prior knowledge [14,15,16]. Therefore, the quality of the contact with the mothers can also affect the intensity of their future gregariousness as adults and the quality of their relationship with conspecifics [17,18].

Farm animals, such as ewes, goats, cows, and buffaloes, tend to isolate themselves from the herd a few hours before giving birth, facilitating the early relationship with their young [19] and avoiding interferences from other adult females during the sensitive period. However, in buffaloes, there have been reports of the existence of communal rearing, including allo-suckling [20,21], so that the role played by the mother could be at least partially replaced by other females. Due to the lack of studies relating imprinted bonding to the corresponding underlying neurological pathways [22], the objective of this review is to summarize and discuss current knowledge on the imprinting process, focusing on buffaloes, and identify welfare-related problems triggered during this sensitive period. In addition, environmental factors, and their effects on the development of the subsequent mother–young bond will be investigated, with a focus on buffaloes.

### Strategies of Maternal Care

The behavior of the mother varies among species [23,24,25]. Females that live and reproduce in large, high-density groups, where the risk of misdirecting maternal care is high, need selective strategies to restrict care exclusively to their own young [26].

In species such as water buffalo, group rearing has been reported as a strategy to support all young [20], including young suckling from alien mothers [21].

In the so-called *altricial species* (e.g., canids, felids, most rodents, and lagomorphs) the mother builds a nest or looks for a protected area in which she gives birth to undeveloped offspring. In these species where the litter has limited sensory and musculoskeletal capabilities, the mother–young bond can take days or even weeks to develop [27]. In other species, called “*carried or housed by the mother*”, the sensory systems of newborns are functional, but thermoregulation is inefficient—as in pigs—and their locomotor capacities are limited.

Conversely, *precocial species*, such as farmed ruminants, are characterized by the birth of a fully developed newborn that follows the mother shortly after parturition, and the mother–offspring relationship develops from the time of parturition and immediately after it to increase the probability of the survival of the young (i.e., by promptly escaping from predators by following the mother) [28,29].

When passing through the birth canal, the young of most farmed ruminants stimulate mechanoreceptors located in the cervico-vaginal region. This triggers the Fergusson reflex, sending information through the spinal cord to the hypothalamus, releasing oxytocin [30]. In addition to stimulating contractility along the birth canal, this hormone acts on the mother’s olfactory bulb, which in turn allows the secretion of dopamine, initiating the sensitive period during which the mother identifies her own offspring [31].

In precocial species, the newborns raise their heads soon after birth, and take a ventral–sternal position, followed by hesitant and sequential attempts to stand, first using the thoracic limbs and then the pelvic limbs. These movements allow the newborn to access the udder and feed [32]. Most farmed ruminants stand up within the first minutes after birth and suckle within the first two hours, finding the udder by exploring the lower part of the mother’s body, guided by various signals originating from the mother’s body. In particular, the newborns are attracted by smooth (e.g., without fleece or coat) and warm surfaces, such as the udder skin [33]. Mothers generally help the newborn by arching the back and flexing one of the hind limbs and adopting a parallel opposite direction position to facilitate access to the nipple. The young quickly learn the location and shape of the udder. This is an example of learning based on the dynamics of the mother–calf interaction and especially on the reinforcement of nursing as a positive reward [34].

In general, the offspring stand and suckle shortly after birth [26]. Lanzoni et al. [22] found that Italian Mediterranean buffalo calves took on average 212.0 ± 110.0 min to suckle, and this behavior was mainly shown during the first six hours. Furthermore, they also found that calves that took more time to stand also took more time to make the first suckling attempt, results that could be related to the calf’s vitality. During this period, the offspring can perceive olfactory, acoustic, visual, and tactile signals from their mothers and the environment [35,36], and develop a lasting mother–young relationship supported by the learning capacity of the newborns [37].

Depending on ecological and environmental factors, buffaloes behave as hiders, along with goats and beef cattle, whereas sheep tend to be followers; thus, the newborns of hider species are kept hidden for several days postpartum, and mothers return intermittently to the site of parturition to nurse their offspring [38]. Although a prompt and clear reciprocal recognition between mother buffalo and calf occurs, different behavioral patterns can be observed. Some cows, in addition to nursing their own calf, allow other calves to suckle. Similarly, some calves only suckle from their mothers, while others try to suckle from alien cows. Generally, those cows accepting alien calves also produce more milk, possibly exceeding the needs of their own calves [39]. This behavior has been observed more frequently in low-birth-weight calves, suggesting that allo-suckling might be a compensatory behavior in neonates with this condition [40]. These calves then drink surplus milk [41] and receive potential immunological benefits [42]. This behavior does not appear to have any negative effect on the progeny of the allo-nursing dams, possibly because they devoted to alien calves only the milk not ingested by their own calf [37]. The sex of the calves can influence this behavior, and therefore, their weight gain. Paranhos da Costa et al. [43] observed that during the first four months of life, male buffaloes presented higher mean daily weight gain and devoted more time to both suckling from their own dams and in communal feeding, compared to female calves. However, although the development of communal nursing is not prevented, buffalo cows and calves develop an imprinted bond allowing mutual recognition and selective attachment [37]. The fact that some buffalo cows allow alien calves to feed has been considered either an altruistic behavior [20], or as a means of eliminating milk that was not ingested by their own young [44]. Unfortunately, the lack of studies in this species does not allow us to determine with greater certainty the causes, costs, and benefits of allo-suckling.

## 2. Sensory Stimulation and Communication during the Imprinting Process

During the sensitive period, numerous sensory communication channels are deployed to ensure mutual recognition between the mother and her offspring [27,45].

Learning is a complex process that involves the acquisition, storage, and retrieval of information, all of which form memory, and consolidate learned behavior [5,46]. The only necessary condition between the association of a stimulus and a response is the close temporal relationship between them. According to Knudsen [47], learning that occurs during the sensitive period has a long-lasting influence on the development of the individual’s social and emotional behavior. During this sensitive period, most newborns have developed vision, hearing, smelling and even touching, in such a way that perhaps imprinting is triggered by the dominant sense of the species at that time [5,6].

Once this link is established, the mother becomes very selective and actively rejects any other unidentified newborn that attempts to suckle [23,48]. In cattle, maternal vision and smell are fundamental factors in establishing the cow–calf bond [49]. In dairy cows, contact for 5 min with her calf immediately after calving is sufficient for the formation of a strong and specific bond between the mother and her calf [50,51]. Although smell is referred to as the most important sensory channel in sheep [52,53] at the beginning of maternal behavior, this cannot be generalized to all species.

### 2.1. Tactile Communication

Tactile communication involves as main recognition factor the physical contact between individuals, which can even occur due to thermo-reception, as in human babies, sheep, puppies, and piglets. These human and nonhuman animals localize and then identify the mother’s udder/nipple to suckle based on thermo-receptors [54,55].

Licking is essential for the development and strengthening of the mother–calf bond in mammals [56]. The cow usually begins liking her calf from the head, perhaps because removal of the fetal membranes decreases the risk of suffocation. Removal of the amniotic fluids can also help to dry the newborn’s coat, thus reducing heat loss, and stimulating nipple-seeking activity [26], while also allowing the mother to learn the specific odor of her own neonate [57].

In Murrah and Surti buffalo immediately after birth, the mother stands up [32], licks and sniffs her calf [58], stimulating respiration, circulation, urination, and defecation of the calf [32]. Multiparous mothers spent more time licking their calves than primiparous, which suggests a higher degree of maternal care expressed by more experienced mothers [58]. In Italian Mediterranean buffalo the dams mainly groomed the calf during the first six hours after calving, to ensure the formation of the cow–calf bond; thereafter, the behavior decreased over time [22]. Both in cattle and buffalo, cows ingest the fetal membranes during this process of conditioning and cleaning of the offspring, whereas sheep and goats normally do not express this behavior [24,26]. Touching the offspring’s face strongly activates the newborn’s oral examination and orientation movements. However, the intensity of the responses depends on the characteristics of the stimulus. For example, lambs respond preferentially to warm, soft surfaces, without the presence of wool [24]. However, in sheep, after the sensitive period, the ewes almost never groom their lambs [59], whereas grooming is quite common in cattle even in adults [60].

### 2.2. Auditory Communication

Auditory imprinting relies on mother–offspring vocal communication and allows acoustic recognition. The vocalizations emitted by the newborns stimulate care and are important components in the regulation of early interactions in many species, for example, humans [61], bats [62,63], and guinea pigs [64]. Sophisticated recognition strategies have been studied in many social mammals where mother and offspring use vocalizations to find and recognize each other even after long periods of separation [65,66,67,68].

Acoustic recognition relies on the one hand on individual distinct vocalizations by the sender and on the other hand on the ability of the receiver to recognize these individually distinct vocalizations. It may also be uni- or bidirectional [65].

In farmed ruminants, the newborns vocalize a few hours after birth to attract the attention of the mother when they need to be fed or protected [32,58]. In sheep, the mother shows an intense peak of vocal activity during the first three hours after parturition [24]. This intense vocal activity may help the newborn to recognize the mother’s voice. After this period, the number of vocal events gradually decreases up to 24 h postpartum [69]. In cattle and sheep, auditory recognition tends to be bidirectional between the mother and her offspring, varying with age [68], breed [70], and time elapsed from bond formation [71].

### 2.3. Olfactory Communication

Olfactory imprinting may be considered the most selective tool used by mothers to recognize their own newborn [72,73]. Immediately after parturition, several species of ungulates, including farmed ruminants such as cattle, buffaloes, goats, and sheep are very receptive to the smell of their young [74]. In these animals, soon after parturition the amniotic fluid is very attractive for the mother and stimulates licking, facilitating the acquisition of chemical signals from the newborn and its acceptance [75,76]. Ewes need two to four hours postpartum to acquire these chemical signals, recognize their lambs and become selective, rejecting all other lambs [77,78]. In a few days, lambs learn to walk ahead of their mothers to be recognized and then take the inverse-parallel position to be definitively identified by their mothers based on the smell originated from the perianal region. Ewes lick the perianal region more frequently than any other part of the lamb’s body [24,79]. Some authors suggest that in this species the vomeronasal organ plays a role in the recognition of the chemical substances emanating from the lamb [80,81]. In small ruminants, smell ceases to be efficient at distances greater than 0.25 m [82,83] requiring that ewes and lambs stay close to each other during the sensitive period.

During the early postpartum period, cell proliferation occurs in specific areas of the mother’s brain, as well as in the main olfactory bulb. The maternal brain is modified by remodeling neural circuits, especially olfactory structures. This process is called adult neurogenesis, a type of brain plasticity that could constitute an adaptive response to motherhood [53] and could facilitate the development of an olfactory memory. Therefore, it seems that the ability of the olfactory system to generate new interneurons plays an important role in the acceptance and recognition of the offspring during the sensitive period. Furthermore, interactions with the newborn lamb will accelerate the maturation of these new neurons in the maternal olfactory bulb [24,53,84].

### 2.4. Visual Communication

In buffalo, the information on visual communication and cow–calf recognition is nil, even though visual cues are commonly used by domestic animals including ruminants, horses, and dogs. Newborns recognize and follow the object or movable entity that provides emotional support, nutrition, and protection. Keller et al. [48] reported that in sheep, the ability to efficiently to recognize their lambs by sight at a distance is higher in multiparous than primiparous ewes. For this reason, it is suggested that the maternal experience has a differential effect on the dynamics of these learning processes.

In summary, it can be hypothesized that, once the maternal–filial bond has been formed, visual and auditory signals are used by mothers and young to orientate and localize the partner as well as for preliminary recognition at a distance, whereas smell is used for the definitive identification, which is performed when the pair is in proximity.

**Figure 1:**
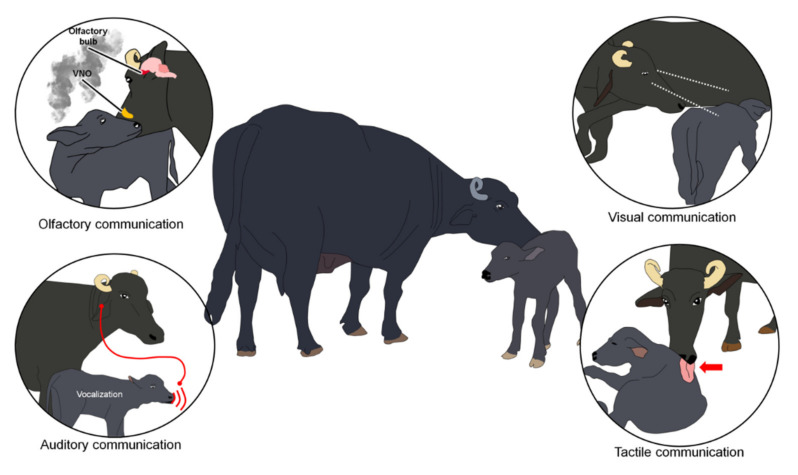
Senses and site of action in the brain during the imprinting process (VNO: vomeronasal organ).

Figure 1 illustrates the different senses and sites of action in the brain during the imprinting process (VNO: vomeronasal organ).

## 3. Neurobiological Mechanism in the Sensitive Period

Imprinting occurs in several stages, within different brain structures, and the consequent release of hormonal cascades allows the development of early learning [85]. Imprinting involves the transmission of the activation stimulus perceived by the senses and a neuronal modification allowing the individual to acquire, store, and retrieve information [2].

In auditory and visual imprinting, the main site of action is the dorsal region of the cerebral hemispheres or roof of the forebrain, especially the left side in chickens [46,86], which would correspond to regions of the cortex in mammals, possibly the prefrontal and cingulate areas [87]. These regions receive input from the primary sensory areas in the anterior region of the brain [87].

In equines, canines, felines, and rodents, in which taste or licking are important, the hippocampus plays a central role. The hippocampus belongs to the limbic system, located on the sides of the thalamus and key to the development of autonomic and endocrine responses, unconscious memory, and the regulation of emotional states [88,89].

The nucleus accumbens is another structure related to imprinting, which plays a key role in achieving positive emotions that involve incentives or motivational aspects of social interaction [90]. This motivational system has neurobiological bases in the mesocortic-limbic dopaminergic pathway, and it is a mechanism that triggers motor activity driving the animal to seek sources of gratification, such as suckling and grooming [91]. It is important to mention that within this midbrain dopaminergic system there are subgroups in the ventral integument area, projecting into the limbic system which includes the olfactory bulb, olfactory tubercle, amygdala and nucleus accumbens [90,91,92,93].

## 4. Neurotransmitters Involved in Imprinting

Imprinting involves communication within the nervous system through neurotransmitters that induce morphological alterations (neuronal plasticity) creating new connections as the offspring learns [94]. There are specific neurotransmitters that are essentials in the development of the mother–young bond.

During parturition, estrogens and oxytocin are released [95]. However, after a few hours, the concentration of these hormones decreases. If recognition did not occur during this period, the bond may not be properly established [37].

Estrogens are mainly female steroid sex hormones, produced by the ovaries, the placenta during pregnancy and, to a lesser extent, by the adrenal glands [96]. The possibility of inducing maternal behavior in non-pregnant ewes by injections of ovarian steroids has been investigated by Le Neindre et al. [97], who found clear evidence of the establishment of selective maternal behavior shown by the treated ewes. Other researchers have found that intracerebroventricular injections of oxytocin also induce maternal responses in estrogen-treated females. However, as for vagino-cervical stimulation, oxytocin is ineffective when given without estrogen priming [26].

Oxytocin is a hormone produced by the supraoptic and paraventricular nuclei of the hypothalamus that is released into the circulation through the neurohypophysis [96]. The role of endogenous oxytocin mediating the onset of maternal behavior has been demonstrated in numerous nonhuman species [98]. Perinatal manipulation of the oxytocin system provides strong evidence for subsequent dysfunctional maternal behaviors [99].

In nonhuman primates, optimal maternal behavior can be altered with an injection of synthetic oxytocin or an antagonist [30]. In the postpartum period, oxytocin can negatively affect anxiety and depression [100]. One of the changes that are believed to be necessary for normal maternal behavior is the decrease in anxiety during the postpartum period [101] allowing the mother to accept more easily an offspring [102].

In the central nervous system, neurons that secrete oxytocin send projections to various sites, including the amygdala, hippocampus, nucleus accumbens, and ventral tegmental area. Oxytocin is released in response to physical and psychological stressors and to various positive or satisfying social stimuli [95]. In both human and animal studies, biological systems that contribute to maternal behavior have been identified, focusing on the oxytokinergic and dopaminergic systems, as oxytocin activates dopamine pathways in response to social cues [103].

Prolactin is a polypeptide hormone synthesized and secreted by cells called lactotropes, located in the adenohypophysis. Although prolactin is best known for its role in milk production, it also plays an important role in maternal care and parental behavior in birds and mammals [104]. During pregnancy, prolactin concentration increases, stimulating neurogenesis in the subventricular zone of the lateral ventricle of the brain. New neurons produced in the forebrain during pregnancy and lactation migrate to the olfactory bulb where they likely participate in processing olfactory cues received by the new mother as she adapts to the needs and challenges of raising young [105]. Therefore, low prolactin levels during early gestation and the consequent suppression of neurogenesis in the brain are associated with increased postpartum anxiety [106].

**Figure 2:**
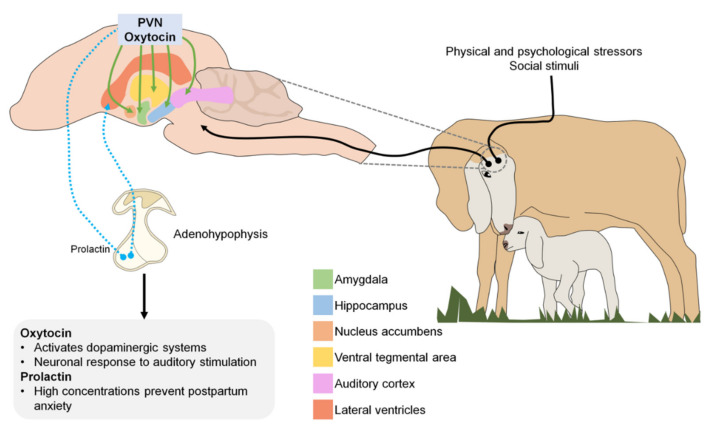
Neurotransmitters involved during the imprinting process (PVN: paraventricular nucleus).

Figure 2 illustrates the different neurotransmitters involved during the imprinting process (PVN: paraventricular nucleus).

## 5. Factors That Interfere with Imprinting during the Sensitive Period

It should be considered that imprinting has a critical stage during which any internal or external condition to the mother and her offspring can compromise not only the mother–offspring relationship, but also the learning process itself and even the well-being and survival of the offspring. External conditions include human interference and some other stimulus from the facility itself or from the natural environment, which can lead to rupture or wrong imprinting [13,15,16].

The negative behaviors of some ungulate mothers, such as sheep, buffaloes and goats, during parturition or lactation include lack of seeking a protected and isolated place to give birth; moving from the birth-site after parturition; brief and insufficient postpartum care; aversion or aggressiveness towards the newborn or abandonment of the newborn [37,107,108].

The abandonment of the newborn could be due to a small investment of time in cleaning the newborn, little vigilance or increase in the distance between the mother and young. In addition, newborn animals with little vocalization frequency or low intensity vocalizations could also induce weak mother–young attachments [109,110,111].

Some of the elements that can interfere with the mother–young sensory exchange can be classified into factors inherent to animals and factors concerning the environment.

### 5.1. Factors Inherent to Animals

Mothers that are inexperienced (primiparous) or in poor physical condition, or with several young may show some inability to recognize and care for more than one newborn. This is accentuated if the mother is moved from her birthplace or has suffered stress or disturbances of any kind despite the significant stimulus generated by the presence of her newborn [112]. For example, primiparous buffaloes are more likely to show inappropriate behavior in comparison with multiparous buffaloes [113,114,115,116]. Inexperienced buffalo cows could sometimes delay the calf´s first suckling with aggressive or rejection behaviors, which can negatively influence the growth or survival of the calf [111]. Similarly, in sheep, mothers with more experience show fewer negative interactions with their lambs, while they show more socio-positive behaviors and maternal care compared to inexperienced mothers. [111,117]. The negative behavior of primiparous mothers may be explained by the lack of experience of previous parturitions, greater anxiety and neophobia due to the presence of the offspring, and a generally more difficult labor [25]. From a physiological point of view the activation of the central oxytokinergic pathways from the first delivery may promote the maternal behavior through an already sensitized system in subsequent parturitions [118]. In relation to the above, it has been documented that those lambs born from primiparous ewes take longer to stand and suckle than the offspring of multiparous ewes [119]. Furthermore, primiparous ewes emit higher vocalizations and spend less time sniffing their lambs, display more locomotor activity, and tend to move when lambs search for the udder, in comparison with multiparous ewes [111].

In addition, some breeds display more maternal behavior than others, establishing the mother–young bond more strongly. For example, Curraleiro Raza Pé Duro cows frequently abandon their offspring to look for water and food, which could disrupt the development of the mother–calf bond during the sensitive period [120], while Dorset horned ewes and some hybrid mothers (Border Leicester X Merino) show greater maternal capacity, staying with their lambs even during human handling. On the contrary, it has been reported that there is a high frequency of abandonment of the offspring by Merino or Romney sheep [111].

Another factor that affects bonding during the sensitive period is the number of newborns per parturition. The abandonment of lambs increases in Merino ewes when they give birth to twins, since when caring for the first lamb, some ewes ignore the second offspring. In fact, in the case of twins, the second lamb receives less licking from the mother than the first one [121]. Furthermore, the weakness of lambs at birth also contributes to the permanent separation of mothers in multiple births [122].

Labor difficulties with prolonged expulsion times increase mortality, since these complications provoke inappropriate behaviors in both the mother and neonate [123]. Mothers with prolonged labor and difficult delivery are often unable to express good care for their offspring due to exhaustion and prolonged pain caused by parturition; consequently, they abandon their newborns more frequently [124]. In addition, difficult and prolonged parturitions produce weak neonates that require more time to stand, reach the udder, and feed successfully [122,124]. The incidence of dystocia in buffaloes is higher when males are delivered, which may be attributed to the higher weight and size of these animals [125], corresponding to a higher mortality rate [126].

### 5.2. Factors Related to the Environment

Any stressing factor either in intensive or extensive production systems, may have direct consequences on the quality of the mother–young bonding. For example, the events that occur before birth and during the first weeks of life may affect the imprinting process, the behavior of the offspring and their adaptation to the environment [127]. In addition, the nutritional level of the pregnant mother may have implications on the weight, vitality and well-being of the newborns, as observed in sheep [128]. Underfed ewes take more time to interact with their lambs, spend less time grooming and show higher levels of aggression towards their lambs [129].

## 6. Conclusions

Imprinting is a process that involves physiological, anatomical, and ethological factors, the alteration of which could lead to survival risks for the offspring. Learning that occurs in this critical period is irreversible and has long lasting effects on the social behavior of the individual. The process is variable across species, but the correct development continues to represent, regardless of the species, a benefit for both the mother and the newborn. Mothers expressing timely and appropriate maternal behaviors, such as bonding and maternal care, represent a key factor for the survival of newborns, particularly those kept in extensive conditions, with a positive impact on farm profitability.

## Data Availability

Not applicable.
